# Time in the therapeutic range, bleeding event, and their determinants in older patients with atrial fibrillation on warfarin in Ethiopia: multicenter cross-sectional study

**DOI:** 10.3389/fphar.2025.1541592

**Published:** 2025-02-13

**Authors:** Zenaw Debasu Addisu, Desalegn Getnet Demsie, Chernet Tafere, Taklo Simeneh Yazie, Destaw Endeshaw, Bereket Bahiru Tefera, Malede Berihun, Dessale Abate Beyene

**Affiliations:** ^1^ Department of Clinical Pharmacy, College of Medicine and Health Sciences, Bahir University, Bahir Dar, Amhara, Ethiopia; ^2^ Department of Pharmacology, College of Medicine and Health Sciences, Bahir Dar University, Bahir Dar, Amhara, Ethiopia; ^3^ Department of Pharmaceutics, College of Medicine and Health Sciences, Bahir Dar University, Bahir Dar, Amhara, Ethiopia; ^4^ Pharmacology and Toxicology Unit, Department of Pharmacy, College of Health Sciences, Debre Tabor University, Debre Tabor, Ethiopia; ^5^ Department of Adult Health Nursing, College of Medicine and Health Sciences, Bair Dar University, Bahir Dar, Amhara, Ethiopia; ^6^ Department of Social Pharmacy, College of Medicine and Health Sciences, Bahir Dar University, Bahir Dar, Amhara, Ethiopia; ^7^ Department of Pharmacy, Debre Berhan University, Debre Birhan, Ethiopia

**Keywords:** older, atrial fibrillation, time in therapeutic range, bleeding events, Ethiopia

## Abstract

**Background:**

Atrial fibrillation (AF) poses significant thromboembolism and bleeding risks, especially in older adults. Warfarin continues to be a primary treatment option, and maintaining the Time in Therapeutic Range (TTR) is critical for ensuring its effectiveness. However, suboptimal TTR is associated with increased risks of stroke, bleeding, and mortality. Despite its importance, there is limited data on warfarin management in Ethiopian older adults with AF. Therefore, this study aimed to determine the TTR, bleeding events, and their determinants, in older patients with AF in Ethiopia receiving warfarin therapy.

**Method:**

In this study, older patients with AF who were treated with warfarin and had follow-up visits between May 2021 and May 2024, and met the inclusion criteria, were included. Patients were categorized based on TTR into two groups: poor anticoagulation (TTR < 65%) and good anticoagulation quality (TTR ≥ 65%). Bivariate and Multivariate Logistic regression was performed to predict determinants of a TTR < 65% and bleeding events. Odds ratios with 95% confidence intervals (CIs) were calculated, and statistical significance was set at P < 0.05.

**Results:**

In this study, 384 patients with AF were included. Of this 53.4% were female. Of these 71% of patients had a TTR below 65%, 29% achieved ≥65%, with a median TTR of 45%. Bleeding events were reported by 13.5% of patients. Poor TTR was significantly associated with age (AOR = 1.199, 95% CI: 1.109–1.297), chronic kidney disease (AOR = 27.809, 95% CI: 7.57–101.76), and infrequent INR monitoring at 31–90-day intervals (AOR = 0.15, 95% CI: 0.004–0.051). Regarding determinants of bleeding events, Patients with diabetes mellitus had a 2.6-fold higher bleeding risk (AOR = 2.585, 95% CI: 1.069–6.250), and a CHA2DS2-VASc score ≥3 significantly increased bleeding risk compared to scores ≤2 (AOR = 7.562, 95% CI: 2.770–20.640).

**Conclusion:**

This study highlights suboptimal warfarin therapy among older Ethiopian patients with AF. Poor anticoagulation was associated with advanced age, chronic kidney disease, and infrequent INR monitoring, while diabetes mellitus and high CHA₂DS₂-VASc scores increased bleeding risks. Close monitoring and frequent INR checks are essential to improving outcomes.

## Background

Atrial fibrillation (AF) is the most common supraventricular tachyarrhythmia and significantly increases the risk of ischemic stroke, particularly in older adults (Marini et al., 2005). A comprehensive review of data on the prevalence, risk factors, complications, and treatment of AF in SSA found that the community-based prevalence of AF is 4.3% in persons aged ≥40 years and 0.7% in people aged ≥70 years, with higher prevalence rates in patients with ischemic stroke, rheumatic heart disease (RHD), and dilated cardiomyopathy (Noubiap and Nyaga, 2019).

Given these alarming statistics, the situation becomes even more concerning when considering Ethiopia’s rapidly aging population. In 2015, Ethiopia’s elderly population numbered 5.2 million, representing over 5% of the total population (Juergens and Galvani, 2019). This figure is projected to increase to 6.1% by 2030 and 10.4% by 2050 (Juergens and Galvani, 2019) which amplifies the healthcare challenges posed by AF and other age-related conditions.

Current guidelines recommend anticoagulation for atrial fibrillation (AF) patients at risk of embolic events (Members et al., 2012). To minimize bleeding risks, warfarin is started at 10%–20% lower doses in older patients (Albabtain et al., 2020a). The European Society of Cardiology (ESC) guidelines advise maintaining a TTR above 70% for optimal vitamin K antagonist (VKA) therapy. However, elderly patients are at higher risk for both thromboembolism and bleeding, even within the therapeutic anticoagulation range (Hindricks et al., 2021; Wieloch et al., 2011). This increased risk is attributed to several factors, including polypharmacy, renal failure, hypertension, and reduced functional status, which raises the likelihood of falls (Bungard et al., 2000; Focks et al., 2016).

The effectiveness of warfarin therapy is measured by the duration patients remain within the therapeutic INR range of 2–3, as a higher TTR reduces hemorrhage and thromboembolism (Habet, 2021). The Global Anticoagulant Registry in the FIELD–Atrial Fibrillation (GARFIELD-AF) study found that patients with a TTR < 65% had a 2.6-times higher risk of stroke and 1.5-times higher risk of severe bleeding (Haas et al., 2016). Since TTR is crucial in assessing the effectiveness and safety of warfarin, measuring it allows clinicians to evaluate the success of the therapy (Habet, 2021).

Previous studies on anticoagulation in older adult AF patients show a median TTR of 52% in Saudi Arabia (Badawoud et al., 2024) and a higher TTR (67%) in those aged ≥65 years in Japan (Marcatto et al., 2016). However, no prior research in Ethiopia has illustrated the TTR, bleeding events, and its determinants in older patients with AF. Therefore, this research aims to determine the TTR, bleeding events, and their determinants, in older patients with AF in Ethiopia receiving warfarin therapy.

Our study contributes to the existing literature in three significant ways. First, examining the TTR, bleeding events, and their determinants are crucial for preventing thromboembolic events and minimizing bleeding risks. Second, this study is particularly relevant given the growing elderly population in Ethiopia and the increasing prevalence of AF, as its findings could inform clinical practices, leading to improved patient outcomes by optimizing anticoagulation strategies. Lastly, the study may offer valuable insights for healthcare providers, policymakers, and researchers, guiding the development of targeted interventions to enhance the management of AF within this population.

## Materials and methods

### Study design, period, and settings

A multicenter cross-sectional study was conducted from May 2021 to May 2024 in three randomly selected teaching hospitals in Amhara National Regional State, Ethiopia: Tibebe Ghion Comprehensive Specialized Hospital (TGCSH), Debremarkos Comprehensive Specialized Hospital (DMCSH), and Hakim Gizaw Hospital (HGH). These hospitals provide tertiary healthcare services and are all affiliated with universities.

### Study population and eligibility criteria

The study included patients aged 65 years and older who had been on warfarin therapy for at least 1 month, with a minimum of two recorded INR measurements, and patients who had follow-up visits between May 2021 and May 2024. However, patients with incomplete INR records or corresponding warfarin dosage data, patients who were either switched to or maintained on anticoagulant agents other than warfarin, those with only a single INR reading, and those whose therapy was discontinued due to planned invasive procedures, and patients with mechanical valves were not included in this study.

### Sample size determination and allocation

The sample size was determined using the single population proportion formula, based on the following parameters: a 95% confidence level, a 5% margin of error, and an estimated prevalence of 0.5, due to the lack of previous studies on older patients with AF. This calculation yielded a sample size of 384 participants

$$n=\frac{\left(\;Z\frac{\;a}{2}\right)2\;P.\left(1-P\right)}{d^{2}}=\frac{\left(\;1.96\right)2\;0.5.\left(1-0.5\right)}{{\left(0.05\right)}^{2}}=384$$

where Z denotes the Z-statistic for a 95% confidence interval (1.96), p (population proportion), and d (margin of error).

**FIGURE 1:**
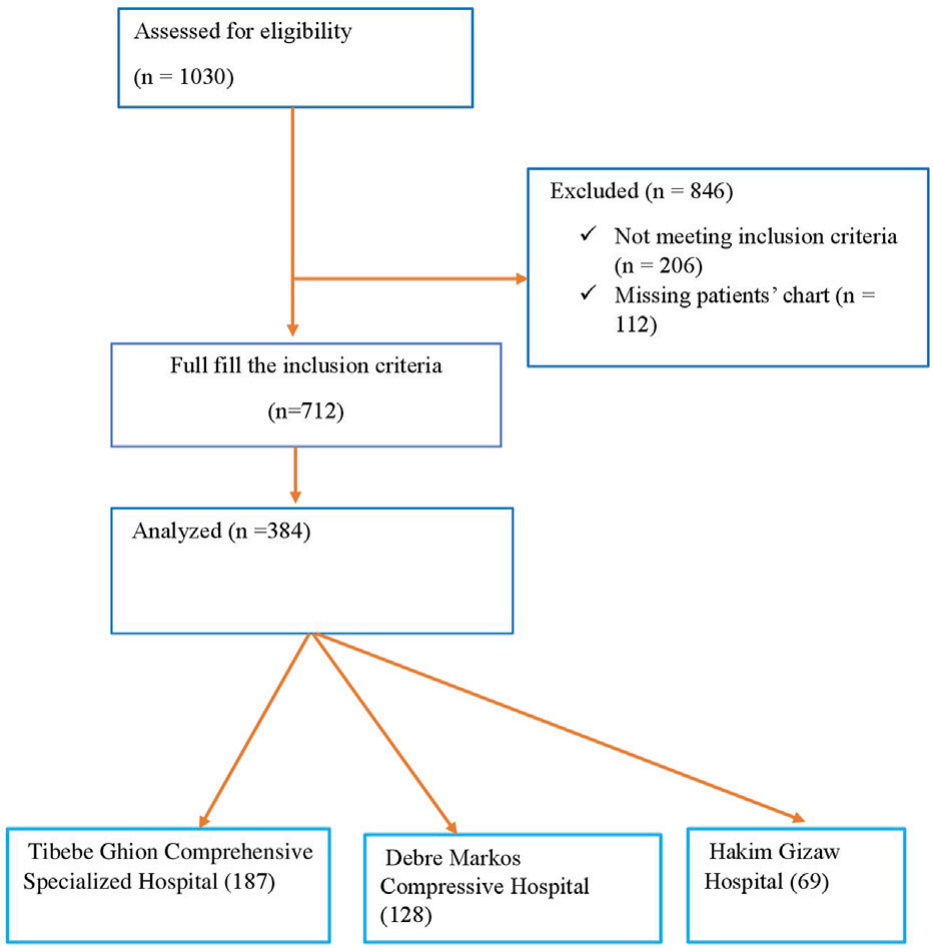
Flowchart showing the selection of older patients with atrial fibrillation from TGCSH, DMCSH, and HGH.

Subsequently, we employed a two-stage sampling technique. First, we randomly selected three hospitals out of the six referral hospitals. Then, we employed a simple random sampling technique to select participants from the chosen hospitals. The total sample size was proportionally allocated for the three selected teaching hospitals (i.e., TGCSH, DMCSH, and HGH) depending on their patient at follow-up. Figure 1 shows a selection of patients diagnosed with AF, from three teaching hospitals.

### Study variables

Quality of anticoagulation, measured as TTR, and bleeding events were considered dependent variables in the study. Independent variables included age, sex, educational status, duration of warfarin therapy, frequency of INR monitoring, concomitant medications interacting with warfarin, presence of comorbidities, concurrent prescription of antiplatelet therapy, number of drugs prescribed, bleeding risk assessed using the HAS-BLED score, thromboembolic risk evaluated using the CHA2DS2-VASc score, and frequency of INR monitoring.

### Data collection instrument and procedure

The TTR of each patient was calculated using the Rosendaal method. The Rosendaal linear interpolation approach is based on the INR-DAY software program (Dr. F.R. Rosendaal, Leiden, Netherlands), which presupposes a linear relationship between two INR measurements and allows the researcher to allocate a specific INR value to each day for each patient (Rosendaal et al., 1993). In short, this method is based on the assumption that the INR value undergoes a broadly linear change when 2 determinations are measured over a given number of days, with the INR being interpolated between the 2 values, i.e., the value increases or decreases by the same amount each day.

### Data quality management

The principal investigator trained three nurses and three pharmacists to ensure a shared understanding of data collection procedures and to familiarize them with the data extraction checklist. Daily on-site supervision and feedback were provided by the principal investigator to maintain the quality of the data collection process. Before starting data collection, the format was cross-verified with available records to ensure alignment. The data collection tool was pre-tested, and necessary modifications were made to enhance its accuracy and relevance. The study questions were rearranged as needed based on pre-test feedback. Incomplete charts were excluded from the analysis. Clarity and consistency were thoroughly reviewed for all completed data collection forms. Regular reviews were conducted to ensure the consistency, completeness, clarity, and accuracy of the data. Any identified errors, uncertainties, or incomplete information were addressed appropriately to maintain the integrity of the collected data.

### Statistical analysis

The data were then entered into the Statistical Package for the Social Sciences (SPSS, IBM Corporation, Armonk, NY, United States) version 25 for the final analysis. Descriptive statistics were used to summarize sociodemographic and other information. Continuous variables were expressed as Means (standard deviations), medians (interquartile ranges [IQRs]), and frequency counts were used to summarize baseline patient characteristics. A chi-square was tested to assess the associations between categorical data. To determine the factors associated with the anticoagulation quality of warfarin, patients were categorized into two groups according to their TTR, namely, poor anticoagulation quality (TTR < 65%) and good anticoagulation quality (TTR ≥ 65%). Binary logistic regression model was used to determine factors associated with the anticoagulation quality of warfarin. Univariate analysis and multivariate logistic regression analysis was used to identify factors associated with poor quality of anticoagulation. Multivariate logistic regression was performed to predict a TTR < 65%.

Additionally, to determine factors associated with bleeding events, patients were divided into two groups: those who experienced and did not. Univariate analysis was initially performed to identify potential predictors, followed by multivariate logistic regression to account for confounding variables.

In univariate analysis, factors associated with TTR < 65% and bleeding events at p < 0.2, along with clinically relevant variables, were included as candidate variables in the multivariate regression models to identify key determinants. Odds ratios (OR) with corresponding 95% confidence intervals (CIs) were calculated. Statistical significance was defined as a bidirectional alpha level of P < 0.05. All statistical analyses were performed using the Statistical Package for Social Sciences (SPSS) version 25.

Drug Interaction Facts (Tatro, 2008), were used to screen patients for a drug-drug interaction between warfarin and other co-prescribed medications.

### Operational definition

• The HAS-BLED score was calculated by assigning 1 point each for the presence of arterial hypertension, renal or hepatic impairment, history of stroke, history of bleeding, labile INR, age ≥ 65 years, and alcohol or drug abuse; 2 points were assigned if both renal and hepatic impairment or alcohol and drug abuse were present. Patients were divided into two groups of bleeding risk based on their overall HAS-BLED score: low risk (score ≤2) and high risk (score ≥3) (Kirchhof et al., 2016).
• The quality of anticoagulation control, measured as the Time in Therapeutic Range (TTR), was stratified into two categories: TTR < 65% and TTR ≥ 65%. Patients with a TTR below 65% were classified as having poor anticoagulation control, while those with a TTR of 65% or higher were considered to have good anticoagulation quality. This cut-off for dichotomized TTR was selected as it is considered to represent the minimal TTR value required for acceptable VKA OAC quality (Crowther et al., 2012).
• Bleeding events were categorized as either major or minor. Major bleeding was defined according to the International Society on Thrombosis and Hemostasis (ISTH) criteria and included fatal bleeding, symptomatic bleeding in a critical organ, bleeding at a surgical site requiring repeat surgery, or bleeding requiring hospitalization (including acute care without an overnight stay). Minor bleeding refers to any other bleeding that does not meet these criteria (Schulman et al., 2005).

## Results

### Sociodemographic characteristics

In this study, 384 charts of patients with AF were reviewed across three hospitals in Amhara regional state. The largest proportion of patients (48.7%) was from TGCSH, followed by DMCSH (33.3%) and HGH (18.0%). The majority of patients were female (53.4%) with a median age of 69 years (IQR 67–74 years). Most patients were married (68.8%) and resided in rural areas (58.9%). Nearly half (43.05%) had no formal education, and the majority were farmers (52.3%), with smaller proportions employed in government roles (16.9%), self-employed (9.8%), or unemployed (20.8%) (Table 1).

**TABLE 1:** Socio-demographic characteristics of study participants.

Variables		Frequency	Percentage	Chi-square p-value
Age, years (median, IQR)	69 (IQR 67–74)			0.000
Sex	Male	179	46.6	0.462
	Female	205	53.4	
Marital status	Married	264	68.8	0.000
	Divorced	9	2.3	
	Widow	111	28.9	
Residence	Urban	158	41.1	0.094
	Rural	226	58.9	
Educational status	No formal education	173	43.05	0.071
	Primary school	121	31.5	
	Secondary school	53	13.8	
	Collage/university	33	8.59	
Employment level	Farmer	201	52.3	0.63
	Government Employed	65	16.9	
	Self-employed	38	9.8	
	Unemployed	80	20.8	
Study site	HGH	69	18.0	1.0
	DMCSH	128	33.3	
	TGCSH	187	48.7	

Key: HGH: hakim gizaw hospital, TGCSH: tibebe gion comprehensive specialized hospital, DMCSH: debre markos comprehensive specialized hospital.

### Clinical characteristics of study participant

In terms of AF type, 271 patients (70.6%) were diagnosed with valvular atrial fibrillation (VAF), while 113 patients (29.4%) had non-valvular atrial fibrillation (NVAF). Regarding comorbidities, the majority of patients had either two (44.5%) or four (33.1%) comorbid conditions. Similarly, in terms of medication use (excluding warfarin), most patients were taking three medications (54.7%), followed by those on two (17.2%) or five or more (17.7%).

The CHA₂DS₂-VASc index, which assesses stroke risk, indicated that 56.0% of patients scored two, 28.9% scored three, and 15.1% scored four. Furthermore, bleeding risk, measured by the HAS-BLED score, showed that 71.1% of patients had a low-to-moderate risk (score ≤2), whereas 28.9% had a high bleeding risk (score ≥3). Regarding bleeding history 86.5% of patients had no history of bleeding, while 13.5% had experienced either minor or major bleeding events (Table 2).

**TABLE 2:** Clinical characteristics of study participant.

Variables		TTR < 65%	TTR ≥ 65%	Total	Chi-square p-value
Types of AF	VAF	170	101	271	0.000
	NVAF	101	10	113	
Number of comorbidities	No	42	12	54	0.000
	One	79	92	171	
	Two	17	10	27	
	Three	33	94	127	
	≥Four	1	4	5	
Number of medications (not including warfarin)	<5medication	170	80	250	0.052
	≥5 medications	103	30	133	
CHA_2_DS_2_-VASc index	Score ≤2	143	73	216	0.017
	Score ≥3	168	38	206	
HAS-BLED bleeding risk score	Score ≤2	105	6	111	0.000
	Score ≥3	12	261	273	
Bleeding history	No bleeding	111	221	332	0.000
	Minor/Major bleeding	44	8	52	

Chronic rheumatic valvular heart disease (CRVHD) was the most prevalent medical comorbidity among the study participants, occurring in 26 (68.2%) patients with AF., this was followed by hypertension, present in 76 (19.8%), and ischemic heart disease, occurring in 63 (16.4%) patients with AF., additionally, participants had various other medical comorbidities, including cardiomyopathy, chronic kidney disease (CKD), diabetes mellitus (DM), stroke, hyperthyroidism, dyslipidemia, hypertensive heart disease, congestive heart failure (CHF), ascites, and asthma (Table 3).

**TABLE 3:** Medical comorbidities of study participant.

Types of comorbidities	Frequency	Percentage
Chronic rheumatic valvular heart disease	262	68.2
Hypertension	76	19.8
Ischemic heart disease	63	16.4
Cardiomyopathy	58	15.1
Chronic kidney disease	48	12.5
Diabetic mellitus	44	11.5
Stroke	37	9.6
Hyperthyroidism	33	8.6
Dyslipidemia	17	4.4
Hypertensive heart disease	16	4.2
Congestive heart disease	13	3.4
Ascites	10	2.6
Asthma	9	2.3
Others*	8	2.08
Deep vein thrombosis	7	1.8

Key: Others* (Tuberculosis, Epilepsy, Arteritis).

**FIGURE 2:**
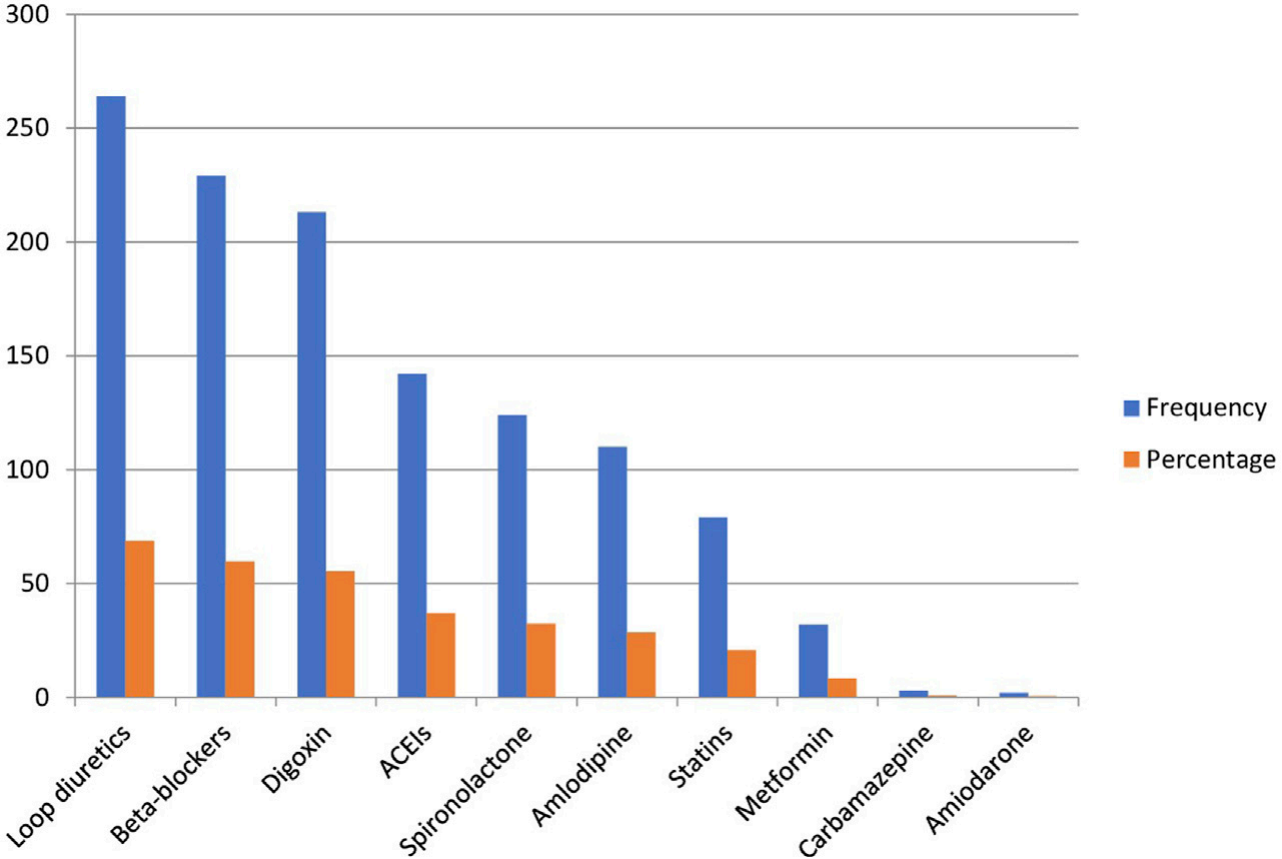
Comedication alongside warfarin in the past 2 months in older patients with atrial fibrillation.

As shown in Figure 2, the majority of study participants, 264 (68.8%), were taking loop diuretics, primarily Furosemide. This was followed by 259 (67.4) participants on digoxin and 229 (59.6%) participants on beta-blockers. Additionally, about one-third of the patients, 142 (37.0%), were using ACE inhibitors, 124 (32.3%) were on spironolactone, and 110 (28.6%) were taking amlodipine. Only two patients (0.5%) were on amiodarone.

### Quality of oral anticoagulation in older patients with atrial fibrillation

#### Percentages of time in the therapeutic range of warfarin

**FIGURE 3:**
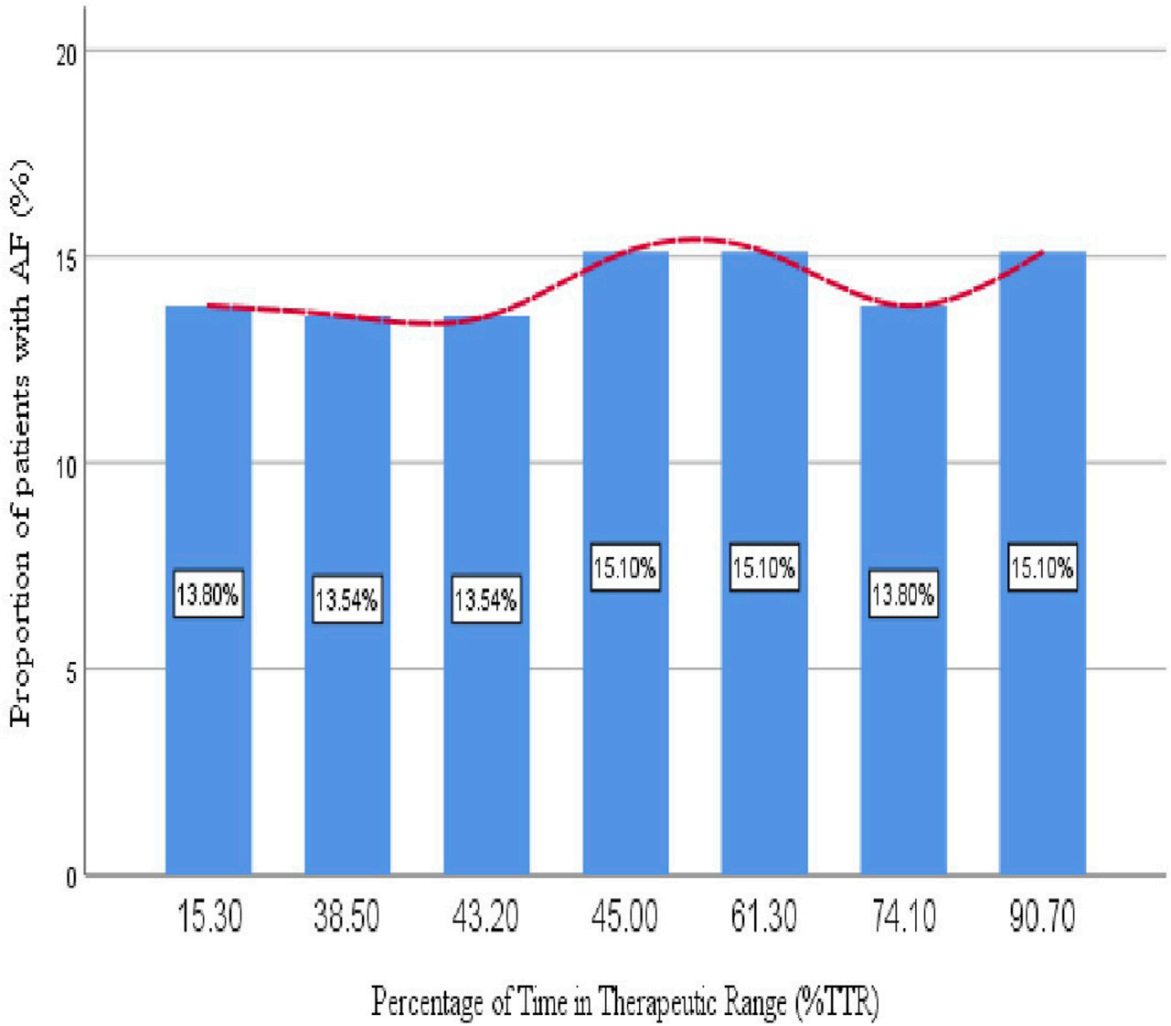
Proportion of older patients with atrial fibrillation relative to the percentage of time in the therapeutic range.

The majority of patients (71%) have a TTR of less than 65%, meaning their INR levels were below the target range most of the time, while only 29% achieved a TTR of 65% or higher, indicating better anticoagulation control in this smaller group. Figure 3 highlights the distribution of AF patients across different TTR ranges, which represent the percentage of time patients’ INR levels remained within the target therapeutic range for anticoagulation. The proportions of patients in each TTR category (15.3%–90.7%) were fairly consistent, ranging from 13.54% to 15.10%. Notably, slight peaks were observed at mid (45.0%) and higher TTR levels (90.7%), both reaching 15.10%, though these differences were minimal.

### Factors associated with poor time in the therapeutic range older patients with atrial fibrillation

Table 4 presents the results of bivariate and multivariate logistic regression analyses identifying predictors of poor TTR in older patients with AF. In the bivariate analysis, the following variables were significantly associated with a TTR of less than 65%: age, dyslipidemia, CKD, previous stroke, INR monitoring frequency of 15–30 days, and INR monitoring frequency of 31–90 days. Variables that were significant in the bivariate analysis were included in the multivariate logistic regression analysis. Findings from the multivariate analysis revealed that older age was a significant predictor of poor TTR, with each additional year increasing the likelihood by 19.9% (AOR = 1.199, 95% CI: 1.109, 1.297, p = 0.000). Additionally, patients with CKD had a 28-fold higher likelihood of poor TTR compared to those without CKD (AOR = 27.809, 95% CI: 7.57, 101.76, p = 0.000). Moreover, Infrequent INR monitoring, particularly at intervals of 31–90 days, was also significantly associated with poorer TTR outcomes compared to more frequent monitoring of less than 7 days (AOR = 0.15, 95% CI: 0.004, 0.0051, p = 0.000). A forest plot illustrating the results of multivariable logistic regression for factors associated with poor TTR is provided as Supplementary Material (Supplementary Figure 1).

**TABLE 4:** Bivariate and multivariate logistic regression analysis results to predictors contributing to poor TTR in older patients with atrial fibrillation (n = 384).

Variable		TTR < 65%	TTR ≥ 65%	COR (95% CI)	P-value	AOR (95% CI)	P-value
Age	Median age 69 (IQR 67–74)			1.105 (1.047–1.168)	0.000	1.199 (1.109, 1.297)	0.000
Dyslipidemia	No	266	101	1		1	
	Yes	7	10	3.762 (1.394, 10.153)	0.009	0.958 (0.45, 2.126)	0.954
Previous stroke	No	250	97	1		1	
	Yes	23	14	0.637 (0.315, 1.289)	0.21	0.719 (0.293, 1.764)	0.471
CKD	No	229	108	1		1	
	Yes	44	3	6.9 (2.101, 22.775)	0.001	27.809 (7.57, 101.76)	0.00
Frequency of INR monitoring	<7 days	36	—	1		1	
	15–30 days	58	45	0.014 (0.003, 0.067)	0.000	0.557 (0.305, 1.017)	0.057
	31–90 days	135	3	0.086 (0.019, 0.379)	0.001	0.15 (0.004, 0.0051)	0.00

Key: AOR: adjusted odds ratio, COD: crude odds ratio, TTR: time in therapeutic range, CKD: chronic kidney disease, INR, international normalization ratio.

### Factors associated with bleeding events in older patients with atrial fibrillation on warfarin

Table 5 summarizes the bivariate and multivariate logistic regression analyses identifying predictors of bleeding events in older patients with AF. In the bivariate analysis, factors associated with bleeding events included DM, type of AF, CHA_2_DS_2_-VASc index, CRVHD, and INR monitoring frequency of 91–180 days. These significant predictors were further analyzed in the multivariate analysis. Findings showed that patients with DM had a 2.6-fold higher likelihood of experiencing bleeding events compared to those without DM (AOR = 2.585, 95% CI: 1.069, 6.250, p = 0.035). A CHA_2_DS_2_-VASc index score of 3 or higher significantly increased the odds of bleeding events, with patients in this category being over seven times more likely to experience bleeding than those with scores of 2 or lower (AOR = 7.562, 95% CI: 2.770, 20.640, p = 0.000). A forest plot illustrating the results of multivariable logistic regression for factors associated with bleeding events is provided as Supplementary Material (Supplementary Figure 2).

**TABLE 5:** Bivariate and multivariate logistic regression analysis results to predictors contributing to bleeding events in older patients with atrial fibrillation (n = 384).

Variable		Bleeding event		COR (95% CI)	P-value	AOR (95% CI)	P-value
		Yes (52)	No (332)				
DM	No	42	341	1		1	
	Yes	10	33	2.157 (0.991, 4.695)	0.053	2.585 (1.069, 6.250)	0.035
Type of atrial fibrillation	NVAF	2	111	1		1	
	VAF	50	221	12.557 (3.000, 52.555)	0.001	4.279 (0.785, 23.316)	0.093
CHA2DS2-VASc index	Score ≤2	47	169	1		1	
	Score ≥3	5	163	9.066 (3.518, 23.367)	0.000	7.562 (2.770, 20.640)	0.000
CRVHD	No	2	118	1		1	
	Yes	50	215	13.850 (3.311, 57.940)	0.000	4.4986 (0.825, 24.381)	0.082
Frequency of INR monitoring	<7 days	0	36	1		1	
	91–180 days	1	31	5.252 (0.701, 39.331)	0.106	1.091 (0.124, 9.597)	0.938

Key: AOR: adjusted odds ratio, DM: diabetes mellitus, COD: crude odds ratio, CRVHD: chronic rheumatic valvular heart disease, NVAF: Non-valvular atrial fibrillation, VAF: valvular atrial fibrillation.

## Discussion

The present study assessed the quality of warfarin therapy among older Ethiopian patients with AF in three teaching hospitals, focusing on TTR, bleeding events, and their determinants. The findings revealed suboptimal anticoagulation control, with a median TTR of 45%, significantly below the recommended threshold of 65% for effective stroke prevention and reduced bleeding risk. The observed TTR ranged from 15.3% to 90.7%. This result is consistent with the Warfarin in Therapeutic Range (WATER) registry in Turkey, which reported a median TTR of 40% among 572 patients with atrial fibrillation (AF) (Turk et al., 2015). However, this result is notably lower than findings from two retrospective cohort studies conducted on older patients in Saudi Arabia, which reported median TTR values of 56.4% and 52%, respectively (Badawoud et al., 2024; Albabtain et al., 2020b). Furthermore, a retrospective cohort study from Japan found a higher TTR among those aged ≥65 years (67% ± 22%) compared to those under 65 years (60% ± 24%) (Marcatto et al., 2016). Moreover, a retrospective study in Turkey, which examined the relationship between TTR and factors such as sociodemographic characteristics, comorbidities, and medication use among warfarin users, reported that (66% of patients experienced poor TTR, compared to 71% in the current study) (Bayyigit et al., 2023).

The discrepancy could partly be explained by differences in study designs, as three studies were cohort-based. In contrast, the current study used a retrospective cross-sectional design, along with variations in patient demographics and clinical characteristics.

In this study, poor TTR levels in warfarin anticoagulation control among older patients with AF were significantly associated with advanced age, CKD, and infrequent INR monitoring, especially when conducted at intervals of 31–90 days. However, other studies have identified additional factors influencing poor anticoagulation control with warfarin. Among Egyptian patients, factors such as female sex, unemployment, illiteracy, and smoking were significantly associated with suboptimal control (Al-shaer et al., 2021). Additionally, heart failure has been reported as a key factor affecting the quality of warfarin therapy in studies conducted in Poland and the United States (Nelson et al., 2013; Ciuruś et al., 2015).

In the current study, older age was a significant predictor of poor TTR, with each additional year increasing the likelihood by 19.9% (AOR = 1.199, 95% CI: 1.109–1.297, p = 0.000). This finding aligns with results from a retrospective study conducted in a tertiary care hospital in Thailand (Lertsanguansinchai et al., 2021). This may be attributed to the presence of multiple comorbidities in older patients, such as CKD and liver disease, as well as poor adherence to managing drug interactions, herbal use, and dietary restrictions, all of which can interact with warfarin and contribute to lower TTR levels.

The present study revealed that patients with CKD were 28 times more likely to have poor TTR compared to those without CKD. This finding is in line with a retrospective cohort study conducted in the United States among patients with newly diagnosed AF, which reported significantly reduced TTR in patients with moderate-to-severe CKD, including those on dialysis, despite similar INR monitoring intensity (Yang et al., 2017). These findings can be explained by the various mechanisms through which CKD impacts TTR. While warfarin primarily metabolizes in the liver, CKD may alter its metabolism by influencing cytochrome P450 2C9 activity (Dreisbach et al., 2003). Additionally, dietary restrictions to manage hyperkalemia and intestinal dysbiosis in CKD can reduce vitamin K intake and production, further affecting warfarin’s efficacy (Guldris et al., 2017).

Regarding with frequency of INR monitoring the College of American Pathologists recommends checking INR at least four times during the first week of therapy, with subsequent monitoring adjusted based on INR stability (Fairweather et al., 1998). For elderly patients with consistently stable INR levels, traditional guidelines suggest routine monitoring every 4 weeks. However, recent evidence indicates that monitoring intervals may be safely extended to 6–12 weeks in select patients who demonstrate sustained stability (Zulkifly et al., 2022; Witt et al., 2009).

In this study, infrequent INR monitoring, particularly at intervals of 31–90 days, was significantly associated with poorer TTR outcomes in older patients with AF compared to more frequent monitoring. The result of this study is in line with several studies highlighting the benefits of frequent INR testing. For instance, a large-scale analysis of over 250,000 INR measurements in patients with chronic AF found a direct relationship between shorter testing intervals and improved TTR (Wieloch et al., 2011). Moreover, research conducted in Veterans Affairs Nursing Homes reported that 99% of INR tests were conducted at 4-week intervals, achieving high-quality warfarin therapy outcomes (Aspinall et al., 2010). Additionally, Patients who use point-of-care technology for self-monitoring their INR levels may achieve better outcomes due to more frequent testing. This promotes greater involvement in their care and decision-making, leading to improved adherence and better management of anticoagulation therapy (Joglar et al., 2024).

Despite this advantage anticoagulation management in Ethiopia faces significant challenges. These include the absence of a dedicated anticoagulation clinic, inadequate patient education strategies, and difficulties in scheduling timely consultations contribute to infrequent INR monitoring, further complicating effective management (Tadesse et al., 2022; Mwita et al., 2024). However, establishing a Pharmacist-Led Anticoagulation Clinic (PLAC) within the hospital could address these issues by improving care accessibility, enhancing patient education, and increasing the frequency of INR monitoring.

The most frequent complication associated with warfarin therapy is bleeding, which can manifest as either minor or major events. In this study, bleeding complications both major and minor were observed in 52 elderly patients with AF. The incidence of bleeding events was approximately 6.77 events per 100 person-years. This result exceeds the gastrointestinal (GI) bleeding rate reported in the elderly subgroup analysis of the All-Nippon Atrial Fibrillation in the Elderly (ANAFIE) Registry, which documented 1.92 events per 100 person-years (Yamamoto et al., 2024). Conversely, it is lower than findings from the WATER registry in Turkey, where major bleeding was observed in 29 patients (5.1%) and minor bleeding in 222 patients (38.8%) (Turk et al., 2015). These disparities can likely be explained by differences in sample size, study design, follow-up periods, and the characteristics of the populations studied. Furthermore, the ANAFIE Registry focused exclusively on GI bleeding.

The findings of this study showed that patients with DM and a CHA₂DS₂-VASc score of 3 or higher had significantly increased odds of experiencing bleeding events. This is consistent with previous research, which has indicated that higher CHA₂DS₂-VASc scores are linked to an elevated risk of bleeding in patients receiving anticoagulants (Zulkifly et al., 2022). Additionally, prior studies have demonstrated that bleeding risk is associated with specific patient characteristics, including hypertension, female sex, severe cardiac disease, renal insufficiency, and advanced age (DiMarco et al., 2005). A possible explanation for these findings is that both the CHA₂DS₂-VASc and bleeding scores share common risk factors, such as advanced age (Roldán et al., 2013). Furthermore, the CHA₂DS₂-VASc score includes diabetes mellitus, which is prevalent among elderly patients with AF. This increased risk of bleeding emphasizes the importance of increased monitoring of diabetic patients to ensure safe and effective management of their anticoagulation therapy, as regular monitoring is critical not only for detecting signs of bleeding but also for good TTR.

Previous studies have extensively documented the frequent interactions between warfarin and other drugs. Among these, the interaction between amiodarone and warfarin is well-known, as amiodarone can alter warfarin metabolism, increasing the risk of bleeding (Schein et al., 2016). In this study, only two patients (0.5%) with AF were prescribed amiodarone. This low usage likely reflects a preference for rate control over rhythm control in AF management, particularly for older patients in resource-limited settings (Wyse et al., 2002). Because rate control is simpler, with fewer side effects and less monitoring, whereas rhythm control treatments like amiodarone are more complex (Heist et al., 2011). Although amiodarone prescriptions were minimal, healthcare providers should remain vigilant about its interactions with warfarin.

### Study limitations

This study has some limitations: First, as a cross-sectional study, it was subject to inherent biases, and challenges in organizing patient histories, along with illegible handwriting, were encountered during chart review, potentially affecting the data quality in some instances. Second, some potential predictors that could influence the TTR and bleeding events, such as herbal diets, adherence status, and non-pharmacologic interventions that impact warfarin anticoagulation, were not recorded. Additionally, while the initial sample size was adequate for estimating the prevalence of AF, it may have limited the statistical power for subgroup analyses.

## Conclusion

This study reveals suboptimal warfarin therapy quality among older Ethiopian patients with AF, as evidenced by a median TTR of 45%, significantly lower than the recommended threshold of 65%. Key factors contributing to poor anticoagulation control included advanced age, CKD, and infrequent INR monitoring. Additionally, patients with DM and higher CHA₂DS₂-VASc scores are associated with an increased risk of bleeding events. Therefore, close monitoring of older patients, especially those with CKD and DM, is essential. Moreover, increasing the frequency of INR monitoring is crucial to improving the quality of warfarin therapy and reducing the risks of bleeding in this population. Furthermore, future prospective studies should investigate additional potential predictors, such as herbal use and medication adherence, that might influence anticoagulation control. Given the small sample size, further nationwide studies with larger sample sizes are necessary to enhance the robustness, generalizability, and strength of group comparisons.

## Data Availability

The original contributions presented in the study are included in the article/Supplementary Material, further inquiries can be directed to the corresponding author.

## Abbreviations

AF, Atrial fibrillation; AOR, Adjusted Odds Ratio; CHF, Congestive heart failure; CKD, Chronic kidney disease; COD, Crude Odds Ratio; CRVHD, Chronic rheumatic valvular heart disease; CT, Computed Tomography; DM, Diabetes mellitus; ECG, Electrocardiogram; ESC, European Society of Cardiology; ISTH, International Society on Thrombosis and Hemostasis; NVAF, Non-valvular atrial fibrillation; TTR, Time in Therapeutic Range; VAF, Valvular atrial fibrillation; VKA, Vitamin K Antagonists; WHO, World Health Organization.
