# Supplementary material for: Time in the therapeutic range, bleeding event, and their determinants in older patients with atrial fibrillation on warfarin in Ethiopia: multicenter cross-sectional study
Source: Front Pharmacol. 2025 Feb 13;16:1541592. doi: 10.3389/fphar.2025.1541592 (PMC11864910; doi:10.3389/fphar.2025.1541592)
Supplement: Supplementary file 1 [file Supplementaryfile1.pdf]

Supplementary Material

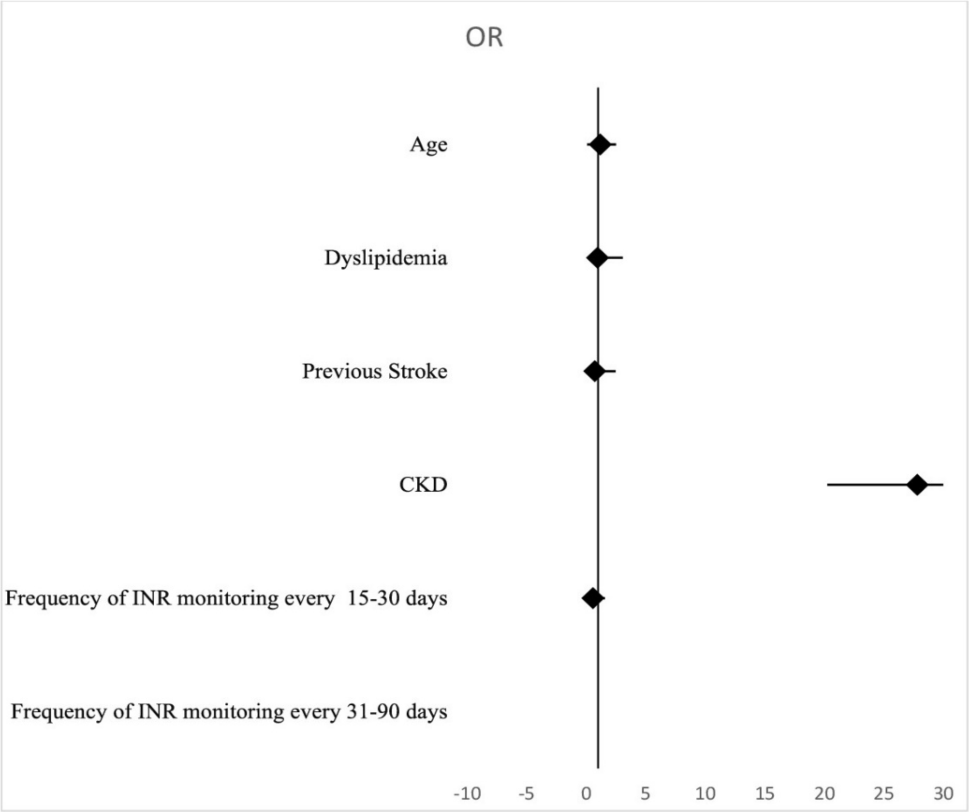

Supplementary Figure 1: Forest plot showing the results of multivariable logistic regression for factors associated with poor time in the therapeutic range in older patients with atrial fibrillation.

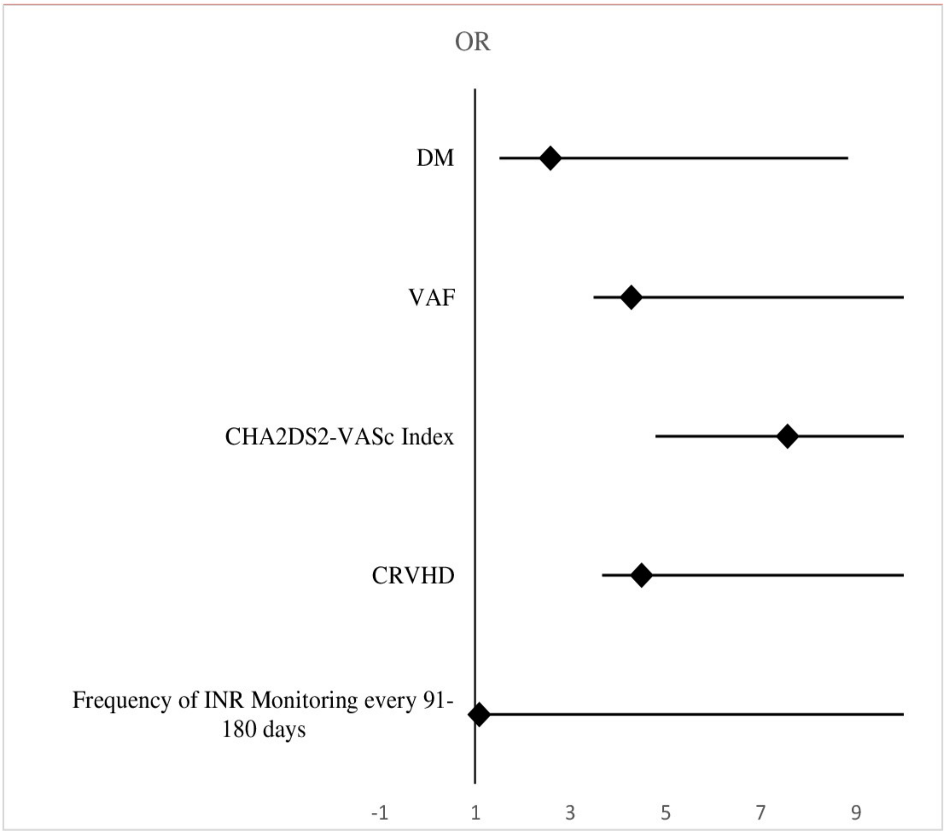

Supplementary Figure 2: Forest plot showing the results of multivariable logistic regression for factors associated with bleeding events in older patients with atrial fibrillation
